# On the antimagicness of generalized edge corona graphs

**DOI:** 10.1016/j.heliyon.2024.e24002

**Published:** 2024-01-05

**Authors:** Nivedha D, Devi Yamini S

**Affiliations:** Vellore Institute of Technology, Chennai, Tamil Nadu, India

**Keywords:** Graph labeling, Antimagic labeling, Generalized edge corona graphs, Spider graphs, Pan graphs

## Abstract

Given a graph *G*, a function of assigning distinct labels {1,2,...,|E(G)|} to E(G) such that w(a)≠w(b), ∀ a,b∈V(G) is an antimagic labeling of *G* where w(a) indicates the vertex sum obtained by summing up all the labels assigned to the edges incident on the vertex *a*. Let *G*, $H_{i}$, 1≤i≤m be connected graphs such that $E(G)=\{e_{1},e_{2},...,e_{m}\}$. A new graph is constructed from *G*, $H_{i}$, 1≤i≤m by adding all possible edges between the end vertices of $e_{i}$ and $V(H_{i})$, i∈{1,2,...,m}. The resulting graph is called the generalized edge corona of *G* and $\left(H_{1},H_{2},...,H_{m}\right)$ which is denoted as $G⋄(H_{1},H_{2},...,H_{m})$. We prove *G* ⋄ $\left(H_{1},H_{2},...,H_{m}\right)$ is antimagic under certain conditions using an algorithmic approach where *G* has only one vertex of maximum degree three (excluding spider graphs containing uneven legs) and $|V(H_{i})|\geq 2$, i∈{1,2,...,m}.

## 1 Introduction

Any graph G in this work has no self loops and parallel edges. Most of the real life situations can be modeled as a graph problem which has attracted the researchers to work on graph theory. Graph labeling is one of the topics in graph theory which has more than 200 techniques. Graph labeling was initiated by Alexander Rosa in 1967. A graph labeling of *G* is a function from V(G)∪E(G) to Z under restrictions [6]. The origin of antimagic labeling can be traced all the way back to Hartsfield et al. in 1990 [8]. Antimagic labeling has numerous results and still a lot of work is under process.

Let M={1,2,...,|E(G)|}. An antimagic labeling of *G* is a bijection f:E(G)→M and $w(x)=\sum_{e\in E(x)}f(e)$ is distinct for all the vertices x∈V(G) where E(x) denotes the set of incident edges on *x*. A graph which preserves an antimagic labeling is recognized as an antimagic graph. In [8], the following conjectures were proposed and remain open for more than three decades.

**Conjecture 1**[8] All connected graphs but $K_{2}$ is antimagic.

**Conjecture 2**[8] All trees but $K_{2}$ is antimagic.

The Table 1 provides a few existing results on antimagic labeling.

**Table 1:** Existing results on antimagic labeling.

*Graphs*	*Ref.*
- Paths	
- Cycles	
- Wheels	
- Complete graphs	[8]

- Graphs with △(*G*)≥*n* − 3	[17]

- Toroidal grids	
- Higher dimensional toroidal grids	
- Cartesian product of cycle and *k*-regular graph	[15]

- Sequential generalized corona graphs	
- Generalized snowflake graphs	[5]

- *n*-barbell graph *n* ≥ 3	
- Edge corona of bistar graph and *k*-regular graph	
- Edge corona of cycles	[12]

- Binomial trees	
- Fibonacci trees	[14]

Complete *m*-ary trees	[3]
Subclasses of trees	[10]
Caterpillars	[11]
Regular graphs	[2], [4]
Biregular bipartite graphs	[16]
Hexagonal lattice	
Prismatic lattice	[1]

## 2 Motivation and applications

A few graph classes and products of graphs were proved to be antimagic, but the antimagic labeling is yet to be explored for the edge corona product of graphs. So, this motivated us to work on the generalized edge corona graph (denoted by *G* ⋄ $\left(H_{1},H_{2},...,H_{m}\right)$).

There are techniques used in surveillance or security model for various buildings which are based on antimagic labeling of double wheel graph, centreless wheel graph, helm, and regular actinia graphs [9]. A few antimagic graphs such as double wheel, helm, path, web, etc. are used in encryption techniques for the security purpose in data transfer [7].

## 3 Preliminaries

**Figure 1:**
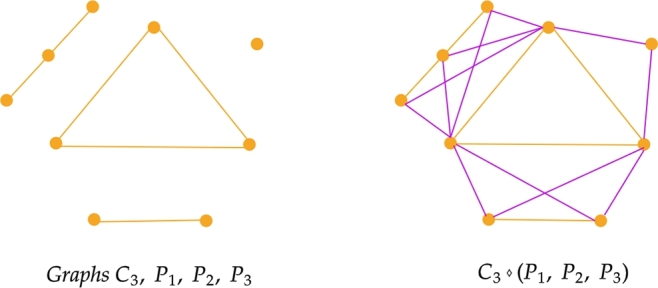
A generalized edge corona of graphs.

The generalized edge corona $G⋄(H_{1},H_{2},...,H_{m})$ is defined as follows:

$$(a)\;V(G⋄(H_{1},H_{2},...,H_{m})=V(G)\cup \overset{m}{\underset{i=1}{⋃}}V(H_{i})(b)\;E(G⋄(H_{1},H_{2},...,H_{m})=\{e_{i}\in E(G):1\leq i\leq m\}\cup \overset{m}{\underset{i=1}{⋃}}E(H_{i})\cup A$$

where *A* denotes the cross edges between each $e_{i}\in E(G)$ and $V(H_{i})$ [13]. An illustration of the generalized edge corona of $C_{3}$ and $\left(P_{1},P_{2},P_{3}\right)$ is given in the Fig. 1.

Let $E^{⁎}(x)$ represent the set of labeled edges incident on *x* for x∈V(G). For a vertex, the degree is the count of incident edges on the vertex. For $v\in V(G⋄(H_{1},...,H_{m}))$, define

$$d(v)=\left\{ \begin{array}{cc} \#\;\text{neighbors of}\;\text{v}\;\text{in}\;\text{G} & \text{for a vertex in}\;\text{G}\\ \#\;\text{neighbors of}\;\text{v}\text{ in }H_{i} & \text{for a vertex in }H_{i} \end{array}\right.$$

and $d^{\prime }(v)=\#\;\text{neighbors}$ of *v* in $G⋄(H_{1},H_{2},...,H_{m})$. In a similar manner, we define the maximum degrees △, ${△}^{\prime }$ and the minimum degrees *δ*, $\delta^{\prime }$. Vertex sum of v∈V(G) is $\sum_{l\in E(v)}f(l)$, denoted by VS; partial vertex sum of v∈V(G) is $\sum_{l\in E^{⁎}(v)}f(l)$, denoted by PVS. A pendant edge attached to a vertex of a cycle is a Pan graph. A spider graph is a tree such that there exist a unique vertex with more than two neighbors and the remaining vertices with less than three neighbors

## 4 Generalized edge corona of graphs

**Figure 2:**
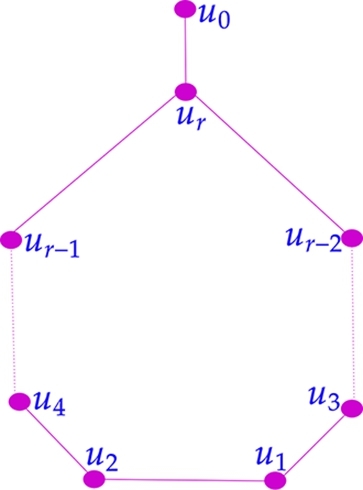
Type I graph *G*_1_.

*G* is connected with a unique maximum degree vertex such that △(G)=3 (except spider graphs containing uneven legs) and the other vertices with degree 2 or 1. Let the graphs $H_{0},H_{1},...,H_{r}$, r≥3 be connected with at least two vertices. Note that the graph *G* can be classified into two types as follows:

**Type I:** A pan graph $G_{1}$ with $V(G_{1})=\{u_{0},u_{1},u_{2},...,u_{r}\}$ where $u_{0}$ is the pendant vertex and $u_{i}$, 1≤i≤r ordered as in the Fig. 2 (the dotted line from $u_{4}$ to $u_{r-1}$ represents a path $u_{4}-u_{6}-u_{8}-...-u_{r-1}$ and the dotted line from $u_{3}$ to $u_{r-2}$ represent a path $u_{3}-u_{5}-u_{7}-...-u_{r-2}$).

**Figure 3:**
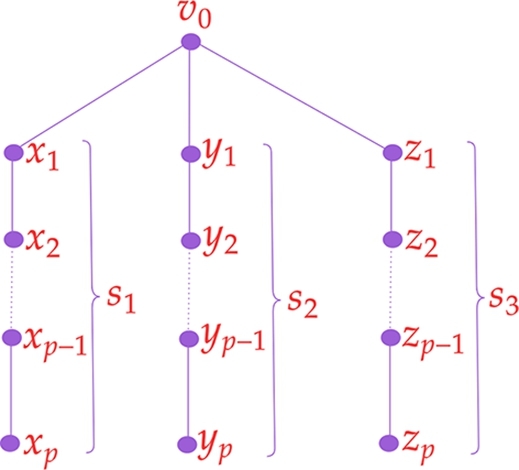
Type II graph *G*_2_.

**Type II:** A spider graph $G_{2}$ with $v_{0}$ as the vertex of degree 3. Let $s_{i}$, 1≤i≤3 be the leg of the spider where each leg represents a path on p≥1 vertices as in the Fig. 3 (the dotted line from $x_{p-1}$ to $x_{2}$ represents a path $x_{p-1}-x_{p-2}-...-x_{2}$, the dotted line from $y_{p-1}$ to $y_{2}$ represent a path $y_{p-1}-y_{p-2}-...-y_{2}$, and the dotted line from $z_{p-1}$ to $z_{2}$ represents a path $z_{p-1}-z_{p-2}-...-z_{2}$). We omit the spider graph with only one vertex of △ as three containing uneven legs from Type II.

### 4.1 $G_{1}⋄(H_{0},H_{1},...,H_{r})$ is antimagic

Note that $G_{1}$ is a Type I graph. Without the loss of generality, let $|V(G_{1})|=|E(G_{1})|=r+1$, r≥3 and $u_{0}u_{r}\in E(G_{1})$. Recall that the graph $H_{i}$, i∈{0,1,2,...,r} is connected with at least two vertices. Let $V(H_{i})=\{v_{1}^{i},...,v_{n_{i}}^{i}\}$ ($|V(H_{i})|=n_{i}$) and $\left|E(H_{i})|=q_{i},\;\forall \;i\in \{0,1,...,r\right\}$. Arrange the graphs $H_{i}$ satisfying the following condition: $\left|V(H_{i})|\leq |V(H_{i+1})\right|$, ∀ i∈{0,1,...,r−1}.

**Construction of** $G_{1}⋄(H_{0},H_{1},...,H_{r})$ **:**

The graph is constructed in the following manner.

$$V(G_{1}⋄(H_{0},H_{1},...,H_{r}))=V(G_{1})\cup V(H_{0})\cup V(H_{1})\cup ...\cup V(H_{r})$$

$$E(G_{1}⋄(H_{0},H_{1},...,H_{r}))=E(G_{1})\cup E(H_{0})\cup E(H_{1})\cup ...\cup E(H_{r})\cup \{u_{0}v_{j}^{0},u_{r}v_{j}^{0}:v_{j}^{0}\in V(H_{0})\}\;\cup \{u_{1}v_{j}^{1},u_{2}v_{j}^{1}:v_{j}^{1}\in V(H_{1})\}\cup \{u_{1}v_{j}^{2},u_{3}v_{j}^{2}:v_{j}^{2}\in V(H_{2})\}\cup \{u_{2}v_{j}^{3},u_{4}v_{j}^{3}:v_{j}^{3}\in V(H_{3})\}\cup ....\cup \;\{u_{r-2}v_{j}^{r-1},u_{r}v_{j}^{r-1}:v_{j}^{r-1}\in V(H_{r-1})\}\cup \{u_{r-1}v_{j}^{r},u_{r}v_{j}^{r}:v_{j}^{r}\in V(H_{r})\}$$

**Figure 4:**
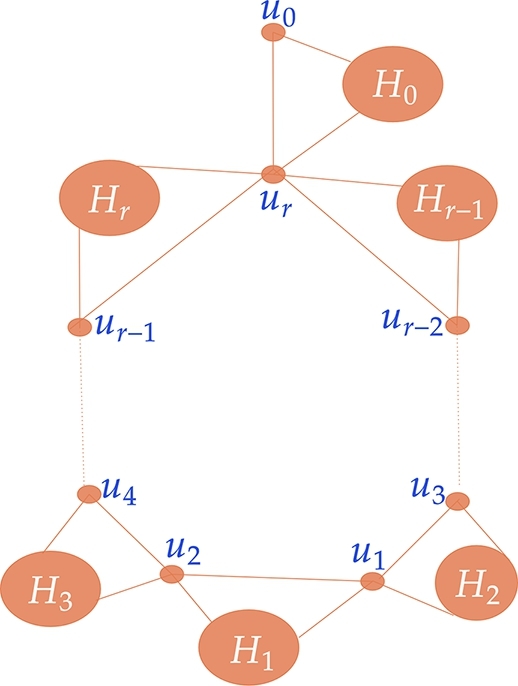
A general representation of *G*_1_⋄(*H*_0_,*H*_1_,...,*H*_*r*_).

The Fig. 4 gives a general representation of $G_{1}⋄(H_{0},H_{1},...,H_{r})$.

### 4.2 Main results

Theorem 4.1 $G_{1}⋄(H_{0},H_{1},...,H_{r})$*,*r≥3*is antimagic subject to the following conditions:*

$$\begin{array}{cc} & △(H_{0})<\delta (H_{1});\\ & △(H_{i})\leq \delta (H_{i+1}),\;\forall \;i\in \{1,2,...,r-1\};\\ & d^{\prime }(u_{0})\leq \delta^{\prime }(H_{i}),\;\forall \;i\in \{0,1,...,r\};\\ & {△}^{\prime }(H_{r})\leq d^{\prime }(u_{1}) \end{array}\}(⁎)$$

Proof We prove that $G_{1}⋄(H_{0},H_{1},...,H_{r})$ is antimagic using an algorithmic approach (Algorithm 1). In each algorithm, the input is the generalized edge corona graph and the output is the antimagic labeling of the graph.

**Algorithm 1:**
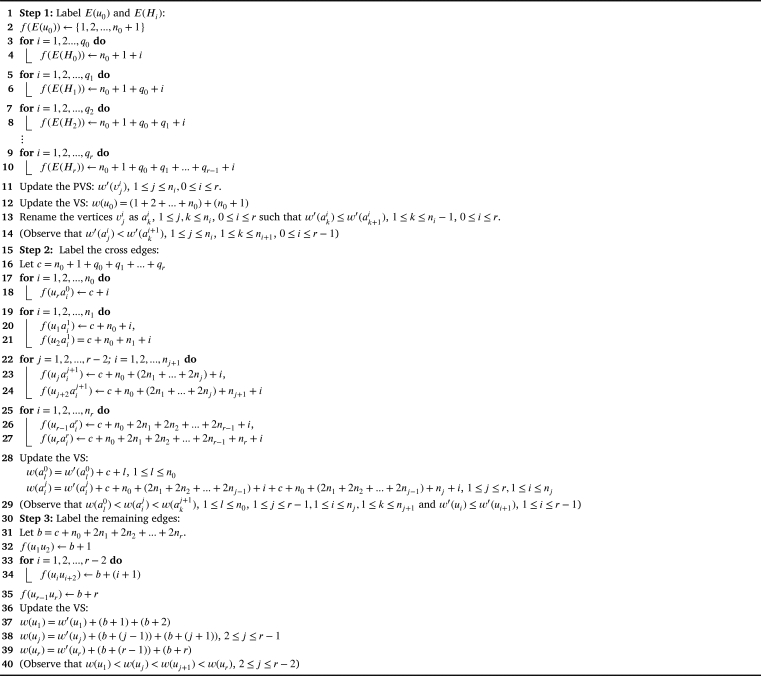
*G*_1_⋄(*H*_0_,*H*_1_,...,*H*_*r*_) is antimagic.

**Proof of distinctness:** In Steps 2 and 3, we have shown the distinctness on the vertex sums $w(a_{j}^{i})$, $1\leq j\leq n_{i}$, 0≤i≤r and $w(u_{i})$, 1≤i≤r respectively. Note that $d^{\prime }(u_{0})\leq \delta^{\prime }(H_{i})$, i∈{0,1,...,r} (using (⁎)). Clearly, $l_{k}\leq l_{s}^{\prime }$ where $l_{k}$ and $l_{s}^{\prime }$ are the labels given to $E(u_{0})$ and $E(a_{j}^{i})$, $1\leq j\leq n_{i},0\leq i\leq r$ respectively. Hence,

(*α*) $$w(u_{0})<w(a_{j}^{i}),\;1\leq j\leq n_{i},\;0\leq i\leq r$$

Let

**Set 1:**$\{c+n_{0}+i|\forall i,\;1\leq i\leq n_{1}$}

**Set 2:**$\{c+n_{0}+2n_{1}+i|\forall i,\;1\leq i\leq n_{2}$}

**Set 3:**{b+1,b+2}

**Set 4:**$\left\{c+n_{0}+2n_{1}+2n_{2}+...+2n_{r-1}+i|\forall i,\;1\leq i\leq n_{r}\right\}$

**Set 5:**$\left\{c+n_{0}+2n_{1}+2n_{2}+...+2n_{r-1}+n_{r}+i|\forall i,\;1\leq i\leq n_{r}\right\}$

**Set 6:**$\left\{(n_{0}+1)+q_{0}+q_{1}+...+q_{r-1}+1,...,(n_{0}+1)+q_{0}+q_{1}+...+q_{r-1}+q_{r}=c\right\}$

Note that ${△}^{\prime }(H_{r})\leq d^{\prime }(u_{1})$ (using (⁎)). All the vertices in $V(H_{r})$ receive d(v) labels of Set 6 where $v\in V(H_{r})$, one of the labels of Set 4, and one of the labels of Set 5 whereas $w(u_{1})$ is the sum of the labels of Set 1, Set 2, and Set 3. Hence,

(*β*) $$w(a_{j}^{r})<w(u_{1}),1\leq j\leq n_{r}$$

Therefore, from (α),(β), Step 2, and Step 3, we get,

$$w(u_{0})<w(a_{j}^{i})<w(a_{k}^{i+1})<w(u_{1})<...<w(u_{r})$$

$(1\leq j\leq n_{i}$, $1\leq k\leq n_{i+1}$, 0≤i≤r−1). Hence, all the vertex sums of the graph $G_{1}⋄(H_{0},H_{1},...,H_{r})$ are distinct. □

**Figure 5:**
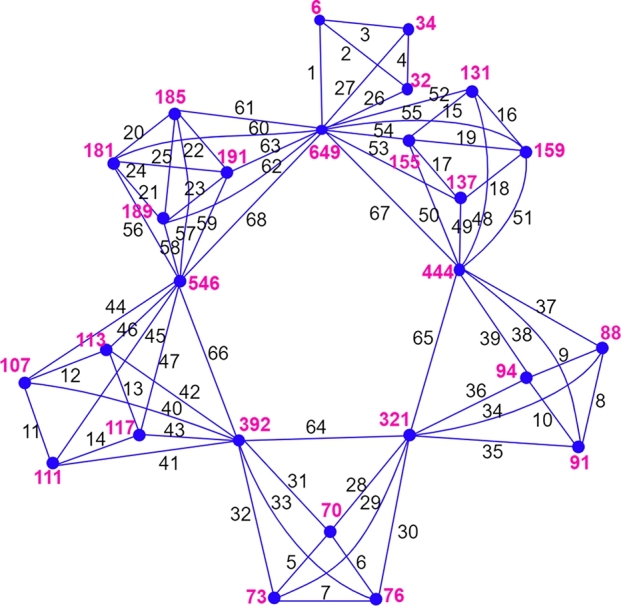
An antimagic labeling of *G*_1_⋄(*K*_2_,*C*_3_,*C*_3_,*C*_4_, a diamond graph, *K*_4_).

An illustration of the above labeling for the graph $G_{1}⋄(H_{0},H_{1},...,H_{5})$ is given in the Fig. 5. Note that $n_{0}=2$, $n_{1}=3$, $n_{2}=3$, $n_{3}=4$, $n_{4}=4$, $n_{5}=4$; $q_{0}=1$, $q_{1}=3$, $q_{2}=3$, $q_{3}=4$, $q_{4}=5$, $q_{5}=6$; and the vertex sums $w(u_{0})=6$, $w(a_{1}^{0})=32$, $w(a_{2}^{0})=34$, $w(a_{1}^{1})=70$, $w(a_{2}^{1})=73$, $w(a_{3}^{1})=76$, $w(a_{1}^{2})=88$, $w(a_{2}^{2})=91$, $w(a_{3}^{2})=94$, $w(a_{1}^{3})=107$, $w(a_{2}^{3})=111$, $w(a_{3}^{3})=113$, $w(a_{4}^{3})=117$, $w(a_{1}^{4})=131$, $w(a_{2}^{4})=137$, $w(a_{3}^{4})=155$, $w(a_{4}^{4})=159$, $w(a_{1}^{5})=181$, $w(a_{2}^{5})=185$, $w(a_{3}^{5})=189$, $w(a_{4}^{5})=191$, $w(u_{1})=321$, $w(u_{2})=392$, $w(u_{3})=444$, $w(u_{4})=546$, $w(u_{5})=649$.

### 4.3 $G_{2}⋄(H_{1},H_{2},...,H_{3p})$ is antimagic

Note that $G_{2}$ is a spider graph (except spider graphs containing uneven legs) on 3p+1 vertices and 3*p* edges containing a vertex $v_{0}$ of △ as 3 and the other vertices with degree 2 or 1 where p≥1. The graphs $H_{1},H_{2},...,H_{3p}$ are connected with at least two vertices where $V(H_{i})=\{v_{1}^{i},...,v_{m_{i}}^{i}\}$ ($|V(H_{i})|=m_{i}$) and $|E(H_{i})|=h_{i}$, ∀i∈{1,2,...,3p}. Arrange the graphs $H_{i}$ satisfying the following condition: $\left|V(H_{i})|\leq |V(H_{i+1})\right|$, i∈{1,2,..,3p−1}.

**Construction of** $G_{2}⋄(H_{1},H_{2},...,H_{3p})$ **:**

The graph is constructed in the following manner.

$$V(G_{2}⋄(H_{1},H_{2},...,H_{3p}))=V(G_{2})\cup V(H_{1})\cup ...\cup V(H_{3p})$$

$$E(G_{2}⋄(H_{1},H_{2},...,H_{3p}))=E(G_{2})\cup E(H_{1})\cup ...\cup E(H_{3p})\cup A\cup B\cup C\cup D$$

where $A=\{x_{p}v_{j}^{1},x_{p-1}v_{j}^{1}:v_{j}^{1}\in V(H_{1})\}\cup \{x_{p-1}v_{j}^{4},x_{p-2}v_{j}^{4}:v_{j}^{4}\in V(H_{4})\}\cup \{x_{p-2}v_{j}^{7},x_{p-3}v_{j}^{7}:v_{j}^{7}\in V(H_{7})\}\cup ...\cup \{x_{1}v_{j}^{3p-5},x_{2}v_{j}^{3p-5}:v_{j}^{3p-5}\in V(H_{3p-5})\}$

$$B=\{y_{p}v_{j}^{2},y_{p-1}v_{j}^{2}:v_{j}^{2}\in V(H_{2})\}\cup \{y_{p-1}v_{j}^{5},y_{p-2}v_{j}^{5}:v_{j}^{5}\in V(H_{5})\}\cup \{y_{p-2}v_{j}^{8},y_{p-3}v_{j}^{8}:v_{j}^{8}\in V(H_{8})\}\cup ...\cup \;\{y_{1}v_{j}^{3p-4},y_{2}v_{j}^{3p-4}:v_{j}^{3p-4}\in V(H_{3p-4})\}$$

$$C=\{z_{p}v_{j}^{3},z_{p-1}v_{j}^{3}:v_{j}^{3}\in V(H_{3})\}\cup \{z_{p-1}v_{j}^{6},z_{p-2}v_{j}^{6}:v_{j}^{6}\in V(H_{6})\}\cup \{z_{p-2}v_{j}^{9},z_{p-3}v_{j}^{9}:v_{j}^{9}\in V(H_{9})\}\cup ...\cup \;\{z_{1}v_{j}^{3p-3},z_{2}v_{j}^{3p-3}:v_{j}^{3p-3}\in V(H_{3p-3})\}$$

$$D=\{x_{1}v_{j}^{3p-2},v_{0}v_{j}^{3p-2}:v_{j}^{3p-2}\in V(H_{3p-2})\}\cup \{y_{1}v_{j}^{3p-1},v_{0}v_{j}^{3p-1}:v_{j}^{3p-1}\in V(H_{3p-1})\}\cup \{z_{1}v_{j}^{3p},v_{0}v_{j}^{3p}:v_{j}^{3p}\in V(H_{3p})\}$$

**Figure 6:**
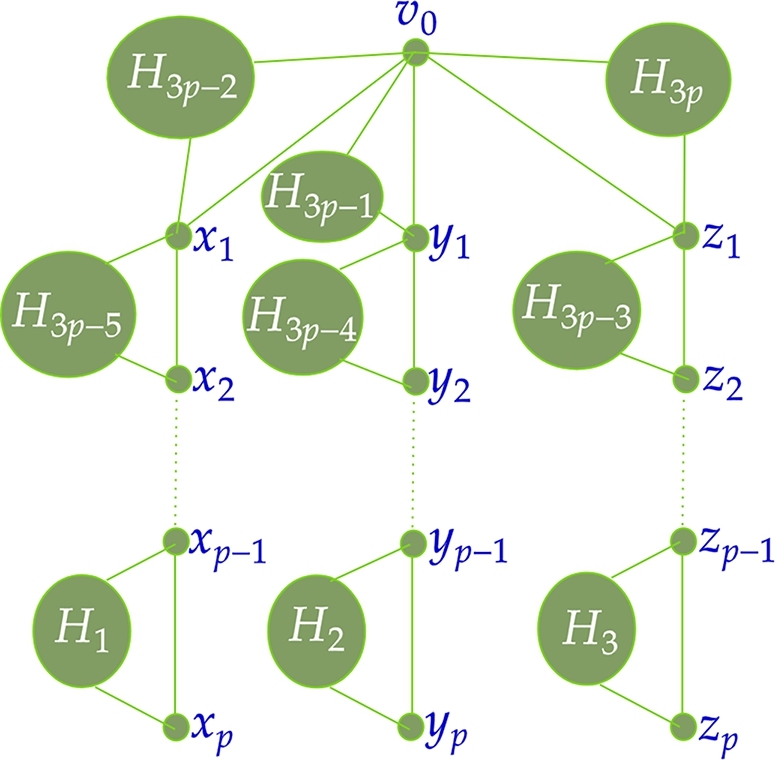
A general representation of *G*_2_⋄(*H*_1_,*H*_2_,...,*H*_3*p*_).

The general representation of the graph $G_{2}⋄(H_{1},H_{2},...,H_{3p})$ is given in the Fig. 6. Note that the graph $G_{2}⋄(H_{1},H_{2},...,H_{3p})$ has $(m_{1}+m_{2}+...+m_{3p})+3p+1$ vertices and $(h_{1}+h_{2}+...+h_{3p})+2(m_{1}+m_{2}+...+m_{3p})+3p$ edges. The following theorem deals with the graph $G_{2}$ as a spider containing legs as a path on at most 2 vertices.

Theorem 4.2 $G_{2}⋄(H_{1},H_{2},...,H_{3p})$*,*p=2*is antimagic subject to the following conditions:*

$$△(H_{i})\leq \delta (H_{i+1}),\forall \;i\in \{1,2,...,5\}d^{\prime }(x_{2})\leq \delta^{\prime }(H_{2}),\;d^{\prime }(y_{2})\leq \delta^{\prime }(H_{3}),\;d^{\prime }(z_{2})\leq \delta^{\prime }(H_{4})$$

*and no restrictions when*p=1*.*

Proof To prove the antimagicness of $G_{2}⋄(H_{1},H_{2},...,H_{3p})$ there are two cases to be discussed: (i) p=1 (ii) p=2.

**Case(i):**p=1

Here $G_{2}$ ≅ $K_{1,3}$. Since, the maximum degree of the graph $K_{1,3}⋄(H_{1},H_{2},H_{3})$ is $|V(K_{1,3}⋄(H_{1},H_{2},H_{3}))|-1$, $K_{1,3}⋄(H_{1},H_{2},H_{3})$ is antimagic (refer Lemma 2.1 in [17]). Hence the proof.

**Case(ii):**p=2

The labeling technique is represented by an Algorithm 2 which is as follows.

**Algorithm 2:**
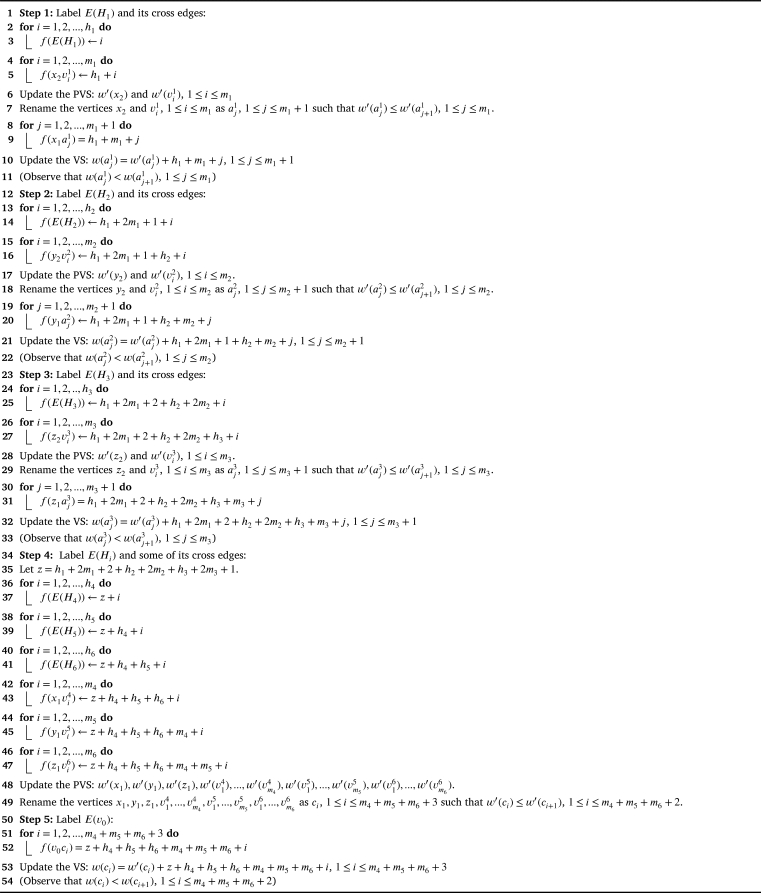
*G*_2_⋄(*H*_1_,*H*_2_,...,*H*_6_) is antimagic.

**Proof of distinctness:** Let $A=h_{1}+2m_{1}+1$ and $B=A+h_{2}+2m_{2}+1$. The condition,

$$d^{\prime }(x_{2})\leq \delta^{\prime }(H_{2}),d^{\prime }(y_{2})\leq \delta^{\prime }(H_{3}),d^{\prime }(z_{2})\leq \delta^{\prime }(H_{4})\text{ leads to }\;d^{\prime }(a_{m_{1}+1}^{1})\leq \delta^{\prime }(H_{2}),d^{\prime }(a_{m_{2}+1}^{2})\leq \delta^{\prime }(H_{3}),d^{\prime }(a_{m_{3}+1}^{3})\leq \delta^{\prime }(H_{4})\text{ respectively.}$$

Note that $w(a_{m_{1}+1}^{1})=A+\sum_{i=1}^{m_{1}}(h_{1}+i)$ and $l_{i}<l_{r}^{\prime }$ where $l_{i}$ and $l_{r}^{\prime }$ are the labels given to $E(a_{m_{1}+1}^{1})$ and $E(a_{j}^{2})$, $1\leq j\leq m_{2}+1$ respectively. Observe that $d^{\prime }(a_{j}^{2})\leq d^{\prime }(a_{m_{2}+1}^{2})$, $1\leq j\leq m_{2}$. Hence,

(1) $$w(a_{m_{1}+1}^{1})<w(a_{j}^{2}),1\leq j\leq m_{2}+1$$

Note that $w(a_{m_{2}+1}^{2})=B+\sum_{i=1}^{m_{2}}(A+h_{2}+i)$ and $l_{i}<l_{r}^{\prime }$ where $l_{i}$ and $l_{r}^{\prime }$ are the labels given to $E(a_{m_{2}+1}^{2})$ and $E(a_{j}^{3})$, $1\leq j\leq m_{3}+1$ respectively. Observe that $d^{\prime }(a_{j}^{3})\leq d^{\prime }(a_{m_{3}+1}^{3})$, $1\leq j\leq m_{3}$. Hence,

(2) $$w(a_{m_{2}+1}^{2})<w(a_{j}^{3}),1\leq j\leq m_{3}+1.$$

Note that $w(a_{m_{3}+1}^{3})=z+\sum_{i=1}^{m_{3}}(B+h_{3}+i)$ and $l_{i}<l_{r}^{\prime }$ where $l_{i}$ and $l_{r}^{\prime }$ are the labels given to $E(a_{m_{3}+1}^{3})$ and $E(c_{j})$, $1\leq j\leq m_{4}$ respectively. Hence,

(3) $$w(a_{m_{3}+1}^{3})<w(c_{j}),1\leq j\leq m_{4}$$

Therefore, from Steps 1,2,3,5, the inequalities (1),(2), and (3), we get

$$w(a_{j}^{1})<w(a_{j+1}^{1})<w(a_{k}^{2})<w(a_{k+1}^{2})<w(a_{l}^{3})<w(a_{l+1}^{3})<w(c_{1})<...<w(c_{m_{4}+m_{5}+m_{6}+3}),$$

($1\leq j\leq m_{1}$, $1\leq k\leq m_{2}$, $1\leq l\leq m_{3}$). Also, $w(v_{0})=\sum_{i=1}^{m_{4}+m_{5}+m_{6}+3}f(v_{0}c_{i})$. Note that the degree and vertex sum of $v_{0}$ will be greater than the degree and the vertex sum of any other vertex in the graph $G_{2}⋄(H_{1},H_{2},...,H_{6})$. Hence, $d^{\prime }(c_{m_{4}+m_{5}+m_{6}+3})<d^{\prime }(v_{0})$ and $w(c_{m_{4}+m_{5}+m_{6}+3})<w(v_{0})$. Therefore, all the vertex sums of the graph $G_{2}⋄(H_{1},H_{2},...,H_{6})$ are distinct. Hence proved. □

**Figure 7:**
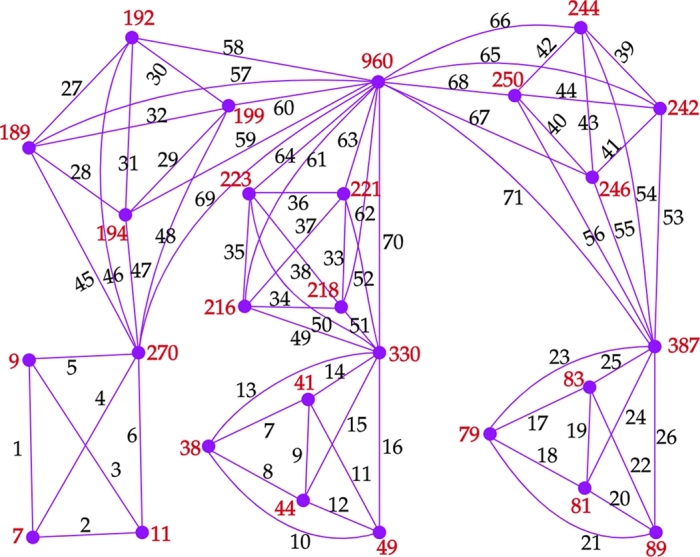
An antimagic labeling of *G*_2_⋄(*K*_2_,*C*_3_,*C*_3_,*K*_4_,*K*_4_,*K*_4_).

An illustration of the above labeling for the graph $G_{2}⋄(H_{1},H_{2},...,H_{6})$ is given in the Fig. 7. Note that $m_{1}=2$, $m_{2}=3$, $m_{3}=3$, $m_{4}=4$, $m_{5}=4$, $m_{6}=4$, $h_{1}=1$, $h_{2}=3$, $h_{3}=3$, $h_{4}=6$, $h_{5}=6$, $h_{6}=6$, and the vertex sums $w(a_{1}^{1})=7,w(a_{2}^{1})=9,w(a_{3}^{1})=11,w(a_{1}^{2})=38,w(a_{2}^{2})=41,w(a_{3}^{2})=44,w(a_{4}^{2})=49,w(a_{1}^{3})=79,w(a_{2}^{3})=81,w(a_{3}^{3})=83,w(a_{4}^{3})=89,w(c_{1})=189,w(c_{2})=192,w(c_{3})=194,w(c_{4})=199,w(c_{5})=216,w(c_{6})=218,w(c_{7})=221,w(c_{8})=223,w(c_{9})=242,w(c_{10})=244,w(c_{11})=246,w(c_{12})=250,w(c_{13})=270,w(c_{14})=330,w(c_{15})=387,w(v_{0})=960$.

Theorem 4.3 $G_{2}⋄(H_{1},H_{2},...,H_{3p})$*,*p>2*is antimagic subject to the following conditions:*

$$\text{(i)}\;△(H_{i})\leq \delta (H_{i+1}),\forall \;i\in \{1,2,...,3p-1\}\text{(ii)}\;d^{\prime }(x_{p})\leq \delta^{\prime }(H_{2}),\;d^{\prime }(y_{p})\leq \delta^{\prime }(H_{3}),\;d^{\prime }(z_{p})\leq \delta^{\prime }(H_{4})\text{(iii)}\;{△}^{\prime }(H_{i})\leq |V(H_{4})|+1,\;\text{where }H_{i}\text{ is adjacent to }z_{1}\text{ and }z_{2}\;\text{(iv)}\;d^{\prime }(z_{2})\leq \delta^{\prime }(H_{i}),\;\text{where }H_{i}\text{ is adjacent to }x_{1}\text{ and }v_{0}$$

Proof We follow the Algorithm 2 till Step 3 by replacing $x_{2},y_{2},z_{2}$ with $x_{p},y_{p},z_{p}$ respectively and $x_{1},y_{1},z_{1}$ with $x_{p-1},y_{p-1},z_{p-1}$ respectively.

**Algorithm 3:**
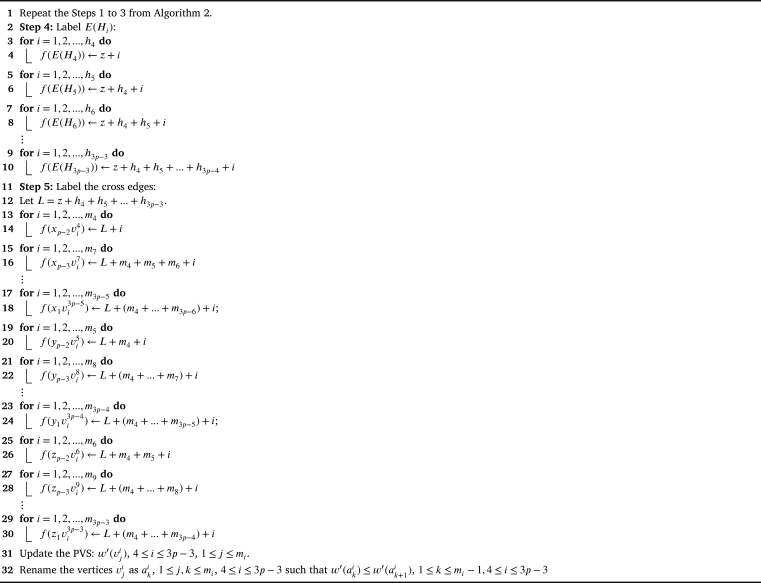

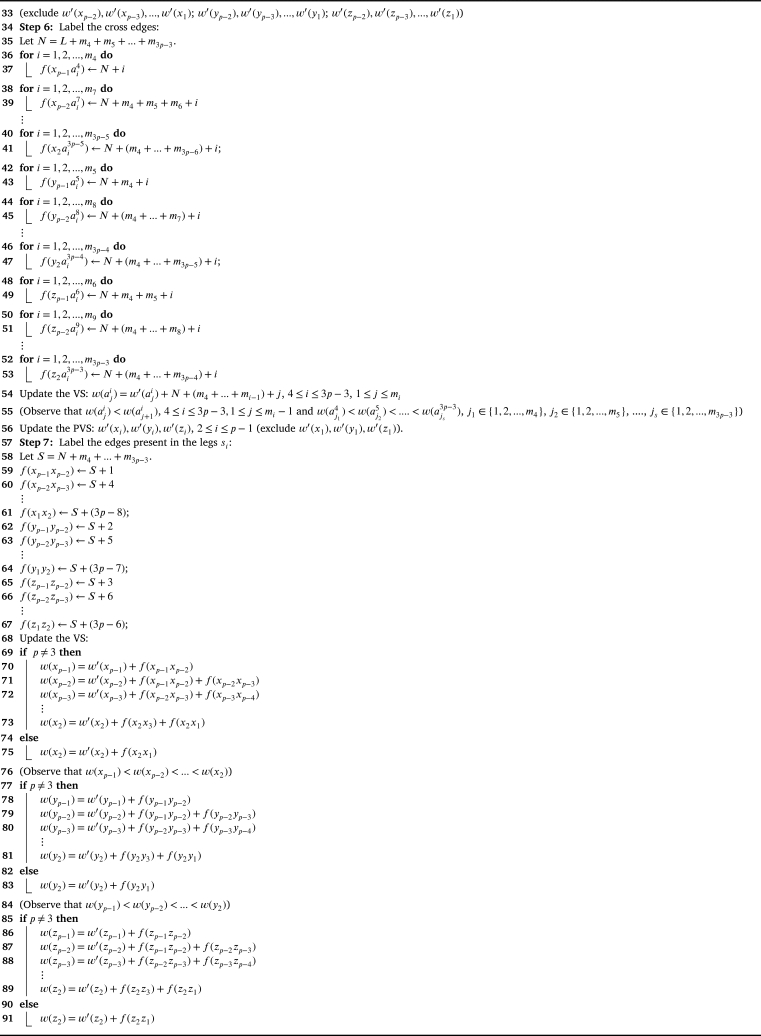

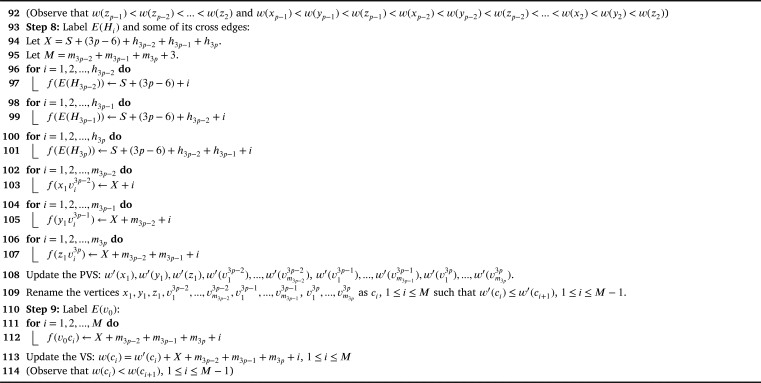
*G*_2_⋄(*H*_1_,*H*_2_,...,*H*_3*p*_) is antimagic, *p* > 2.

**Proof of distinctness:** The condition (ii),

$$d^{\prime }(x_{p})\leq \delta^{\prime }(H_{2}),d^{\prime }(y_{p})\leq \delta^{\prime }(H_{3}),d^{\prime }(z_{p})\leq \delta^{\prime }(H_{4})\text{ leads to }\;d^{\prime }(a_{m_{1}+1}^{1})\leq \delta^{\prime }(H_{2}),d^{\prime }(a_{m_{2}+1}^{2})\leq \delta^{\prime }(H_{3}),d^{\prime }(a_{m_{3}+1}^{3})\leq \delta^{\prime }(H_{4})\text{ respectively.}$$

We follow the same procedure as in the proof of distinctness of Theorem 4.2 to obtain the inequalities (1) and (2).

Note that $w(a_{m_{3}+1}^{3})=z+\sum_{i=1}^{m_{3}}(B+h_{3}+i)$ and $l_{i}<l_{r}^{\prime }$ where $l_{i}$ and $l_{r}^{\prime }$ are the labels given to $E(a_{m_{3}+1}^{3})$ and $E(a_{j}^{4})$, $1\leq j\leq m_{4}$ respectively. Hence,

(4) $$w(a_{m_{3}+1}^{3})<w(a_{j}^{4}),1\leq j\leq m_{4}$$

The condition (iii), ${△}^{\prime }(H_{i})\leq |V(H_{4})|+1$ where $H_{i}$ is adjacent to $z_{1}$ and $z_{2}$, leads to ${△}^{\prime }(H_{3p-3})\leq m_{4}+1$. Observe that $d^{\prime }(x_{p-1})>m_{4}+1$ (since $x_{p-1}$ is adjacent to $V(H_{4})$ and $V(H_{1})$). Hence, ${△}^{\prime }(H_{3p-3})<d^{\prime }(x_{p-1})$. Let

**Set A:**$\left\{z+h_{4}+h_{5}+...+h_{3p-4}+1,...,L\right\}$

**Set B:**$\left\{L+m_{4}+m_{5}+...+m_{3p-4}+1,...,N\right\}$

**Set C:**$\left\{N+m_{4}+...+m_{3p-4}+1,...,S\right\}$

All the vertices in $V(H_{3p-3})$ receive d(v) labels of Set A where $v\in V(H_{3p-3})$, one of the labels of Set B, and one of the labels of Set C whereas $w(x_{p-1})=(S+1)+\sum_{i=1}^{m_{1}+1}(h_{1}+m_{1}+i)+\sum_{i=1}^{m_{4}}(N+i)$. Hence,

(5) $$w(a_{j}^{3p-3})<w(x_{p-1}),1\leq j\leq m_{3p-3}$$

The condition (iv), $d^{\prime }(z_{2})\leq \delta^{\prime }(H_{i})$ where $H_{i}$ is adjacent to $x_{1}$ and $v_{0}$ leads to $d^{\prime }(z_{2})\leq$ $\delta^{\prime }(H_{3p-2})$. Clearly, $l_{i}<l_{r}^{\prime }$ where $l_{i}$ and $l_{r}^{\prime }$ are the labels given to $E(z_{2})$ and $E(c_{j})$, $1\leq j\leq m_{3p-2}$ respectively. Hence,

(6) $$w(z_{2})<w(c_{j}),1\leq j\leq m_{3p-2}$$

Therefore, from Steps 1,2,3,6,7,9, the inequalities (1),(2),(4),(5), and (6), we get

$$w(a_{j}^{1})<w(a_{j+1}^{1})<w(a_{k}^{2})<w(a_{k+1}^{2})<w(a_{l}^{3})<w(a_{l+1}^{3})<w(a_{j_{1}}^{4})<w(a_{j_{2}}^{5})<....<w(a_{j_{s}}^{3p-3})<w(x_{p-1})<w(y_{p-1})<w(z_{p-1})<....<w(x_{2})<w(y_{2})<w(z_{2})<w(c_{1})<...<w(c_{M})$$

($1\leq j\leq m_{1}$, $1\leq k\leq m_{2}$, $1\leq l\leq m_{3}$, $1\leq j_{1}\leq m_{4}$, $1\leq j_{2}\leq m_{5}$, ..., $1\leq j_{s}\leq m_{3p-3}$). Also, $w(v_{0})=\sum_{i=1}^{M}f(v_{0}c_{i})$. Note that the degree and the vertex sum of $v_{0}$ will be greater than the degree and the VS of any other vertex in the graph $G_{2}⋄(H_{1},H_{2},...,H_{3p})$. Hence, $d^{\prime }(c_{M})<d^{\prime }(v_{0})$ and $w(c_{M})<w(v_{0})$. Therefore, all the vertex sums of the graph $G_{2}⋄(H_{1},H_{2},...,H_{3p})$ are distinct. □

**Figure 8:**
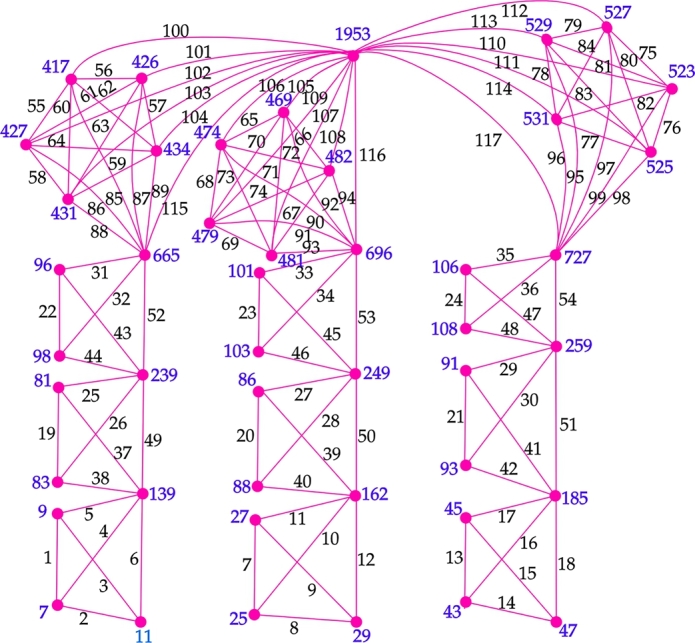
An antimagic labeling of *G*_2_⋄(*K*_2_,*K*_2_,*K*_2_,*K*_2_,*K*_2_,*K*_2_,*K*_2_,*K*_2_,*K*_2_,*K*_5_,*K*_5_,*K*_5_), (*p* = 4).

An illustration of the above labeling for the graph $G_{2}⋄(H_{1},H_{2},...,H_{12})$ is given in the Fig. 8. Note that $m_{i}=2$, i∈{1,2,...,9}, $m_{j}=5$, j∈{10,11,12}, $h_{i}=1$, i∈{1,2,...,9}, $h_{j}=10$, j∈{10,11,12} and the vertex sums $w(a_{1}^{1})=7$, $w(a_{2}^{1})=9$, $w(a_{3}^{1})=11$, $w(a_{1}^{2})=25$, $w(a_{2}^{2})=27$, $w(a_{3}^{2})=29$, $w(a_{1}^{3})=43$, $w(a_{2}^{3})=45$, $w(a_{3}^{3})=47$, $w(a_{1}^{4})=81$, $w(a_{2}^{4})=83$, $w(a_{1}^{5})=86$, $w(a_{2}^{5})=88$, $w(a_{1}^{6})=91$, $w(a_{2}^{6})=93$, $w(a_{1}^{7})=96$, $w(a_{2}^{7})=98$, $w(a_{1}^{8})=101$, $w(a_{2}^{8})=103$, $w(a_{1}^{9})=106$, $w(a_{2}^{9})=108$, $w(x_{2})=139$, $w(y_{2})=162$, $w(z_{2})=185$, $w(x_{3})=239$, $w(y_{3})=249$, $w(z_{3})=259$, $w(c_{1})=417$, $w(c_{2})=426$, $w(c_{3})=427$, $w(c_{4})=431$, $w(c_{5})=434$, $w(c_{6})=469$, $w(c_{7})=474$, $w(c_{8})=479$, $w(c_{9})=481$, $w(c_{10})=482$, $w(c_{11})=523$, $w(c_{12})=525$, $w(c_{13})=527$, $w(c_{14})=529$, $w(c_{15})=531$, $w(c_{16})=665$, $w(c_{17})=696$, $w(c_{18})=727$, $w(v_{0})=1953$.

## 5 Conclusion

Though most of the research focus was on antimagic labeling of general graphs and various products of graphs, there have been no results on antimagic labeling of generalized edge corona of graphs until now. Hence, we were concerned with antimagic labeling of $G⋄(H_{1},H_{2},...,H_{m})$ where |E(G)|=m under certain restrictions. In our future work, we intend to focus on the antimagicness of the spider graphs with the maximum degree three having uneven legs. In addition, we pose the following problem as a future direction of research: ‘For any connected graph *G* with exactly one vertex of maximum degree three, is $G⋄(H_{1},H_{2},...,H_{m})$ antimagic?’

## CRediT authorship contribution statement

**Nivedha D:** Writing – review & editing, Writing – original draft, Validation, Resources, Methodology, Investigation, Conceptualization. **Devi Yamini S:** Writing – review & editing, Validation, Supervision, Methodology, Investigation, Formal analysis, Conceptualization.

## Declaration of Competing Interest

The authors declare no conflict of interest.
